# Dietary Supplement Use Differs by Socioeconomic and Health-Related Characteristics among U.S. Adults, NHANES 2011–2014

**DOI:** 10.3390/nu10081114

**Published:** 2018-08-17

**Authors:** Alexandra E. Cowan, Shinyoung Jun, Jaime J. Gahche, Janet A. Tooze, Johanna T. Dwyer, Heather A. Eicher-Miller, Anindya Bhadra, Patricia M. Guenther, Nancy Potischman, Kevin W. Dodd, Regan L. Bailey

**Affiliations:** 1 Department of Nutrition Science, Purdue University, 700 W. State St., West Lafayette, IN 47907, USA; cowan9@purdue.edu (A.E.C.); jun24@purdue.edu (S.J.); heicherm@purdue.edu (H.A.E.-M.); 2 National Institutes of Health, Office of Dietary Supplements, 6100 Executive Blvd., Bethesda, MD 20892-7517, USA; jaime.gahche@nih.gov (J.J.G.); dwyerj1@od.nih.gov (J.T.D.); potischn@mail.nih.gov (N.P.); 3 School of Medicine, Wake Forest University, Winston-Salem, NC 27101, USA; jtooze@wakehealth.edu; 4 Department of Statistics, Purdue University, 250 N. University St., West Lafayette, IN 47907, USA; bhadra@purdue.edu; 5 Department of Nutrition and Integrative Physiology, University of Utah, 250 South 850 East, Salt Lake City, UT 84112, USA; patricia.guenther@utah.edu; 6 National Institutes of Health, National Cancer Institute, Medical Center Drive, Rockville, MD 20850, USA; doddk@mail.nih.gov

**Keywords:** dietary supplements, nutrients, NHANES, income, SNAP

## Abstract

The objective of this study was to estimate the prevalence of use and types of dietary supplements (DS) used by U.S. adults (≥19 years) by sociodemographic characteristics: family income-to-poverty ratio (PIR), food security status, and Supplemental Nutrition Assistance Program (SNAP) participation using NHANES 2011–2014 data (*n* = 11,024). DS use was ascertained via a home inventory and a retrospective 30-day questionnaire. Demographic and socioeconomic differences related to DS use were evaluated using a univariate *t* statistic. Half of U.S. adults (52%) took at least one DS during a 30-day period; multivitamin-mineral (MVM) products were the most commonly used (31%). DS and MVM use was significantly higher among those with a household income of ≥ 350% of the poverty level, those who were food secure, and SNAP income-ineligible nonparticipants across all sex, age, and race/ethnic groups. Among women, prevalence of use significantly differed between SNAP participants (39%) and SNAP income-eligible nonparticipants (54%). Older adults (71+ years) remained the highest consumers of DS, specifically among the highest income group (82%), while younger adults (19–30 years), predominantly in the lowest income group (28%), were the lowest consumers. Among U.S. adults, DS use and the types of products consumed varied with income, food security, and SNAP participation.

## 1. Introduction

The Dietary Guidelines for Americans (DGA) states that nutrient needs should be met primarily from nutrient-dense foods because, in addition to vitamins and minerals, they contain fiber and other naturally occurring substances with beneficial health effects. The DGA also state that in certain cases, fortified foods and dietary supplements (DS) may be useful [1]. In 2003–2006, about half of adults in the U.S. used at least one dietary supplement daily [2]. Adults at the highest adjusted income have higher micronutrient intakes and lower risk of dietary inadequacy than those with lower incomes [3]. 

The prevalence of food insecurity has increased overtime [4]. In 2016, 40.6 million Americans lived in poverty, an increase from 33.3 million in 2000 [5,6]. Since dietary choices and nutrient intakes are commonly influenced by income, people with lower incomes are more likely to have lower quality, less nutrient-dense diets [7,8]. Nutrient adequacy in adults is related to income [3,9]; however, little is known about DS use patterns according to income indicators among U.S. adults. Therefore, the objectives of this study were to provide updated estimates of DS use and to examine the relationship between DS use and demographic, socioeconomic, and health-related characteristics among U.S. adults, using data from the National Health and Nutrition Examination Survey (NHANES) 2011–2014.

## 2. Materials and Methods 

The NHANES, conducted by the National Center for Health Statistics, is a nationally representative, continuous cross-sectional survey of the noninstitutionalized, civilian residents of the U.S. [10]. NHANES employs a complex, multistage probability sampling design. The NHANES protocol includes an in-person household interview as well as a follow-up health examination in a mobile examination center for each participant. All data presented in this report were collected during the in-person household interview, with the exception of body mass index. Persons who were less than 19 years of age (*n* = 7939), pregnant or lactating (*n* = 184), or had unknown or missing data on the use of dietary supplements (*n* = 4) were excluded, yielding a final analytic sample size of 11,024 U.S. adults. Written informed consent was obtained for all participants or proxies and NHANES survey protocol was approved by the Research Ethics Review Board at the Centers for Disease Control and Prevention, National Center for Health Statistics.

All questionnaire data used for this analysis, including the demographic and lifestyle data on age, sex, race and Hispanic origin, educational attainment, income, smoking status, alcohol use, self-reported health status, and health insurance coverage were collected from participants using the computer-assisted personal interview system during the in-person household interview. In NHANES, race and Hispanic origin is categorized as non-Hispanic white, non-Hispanic black, non-Hispanic Asian, or Hispanic. Age groupings were constructed using the Dietary Reference Intake age categories [11]. Educational attainment was categorized as completion of less than high school, high school diploma or general equivalency diploma, or more than high school. Current health status was classified as excellent/very good, good, or fair/poor. Health insurance coverage of the participant at the time of the survey was categorized as either public, private (including those covered under both private and public plans), or uninsured [12]. Current smoking status was determined based on whether participants were never smokers (smoked < 100 cig/lifetime), former smokers (>100 cig/lifetime but do not currently smoke), or current smokers. Current smokers were then further classified based on whether they smoked cigarettes daily (current, daily) or whether they classified themselves as a smoker, but did not smoke cigarettes daily (current, occasional) [13]. Alcohol consumption was assessed using three questions from the NHANES Alcohol Use Questionnaire that measured use in the last 12 months, frequency, and number of drinks. A standard drink was defined at the time of the interview as a 12 fl. oz. (354 mL) glass of beer, a 5 fl. oz. (148 mL) glass of wine, or 1.5 fl. oz. (44 mL) of liquor [12]. The mean daily drink number was calculated as the number of days a participant reported drinking in the past 12 months multiplied by the usual number of drinks that were consumed divided by the total number of days, and was categorized as 0, 1, 2, or ≥3 drinks/day [12].

DS use in the previous 30 days prior to the household interview was collected via the Dietary Supplement Questionnaire. Trained NHANES interviewers asked the participant about their use of vitamins, minerals, herbals, and other DS. Participants were asked to show interviewers the containers for all products taken in the past 30 days. For each DS reported, interviewers recorded label information including the product name, manufacturer, form (e.g., tablet), and strengths per serving. Participants were also asked about the consumption frequency, dose, and duration of use, for all products reported. Containers were examined for 83% of products reported. If containers were not available, participants were asked to recall in detail the product that they had taken. NHANES nutritionists at the National Center for Health Statistics then matched products reported by participants to product labels, obtained from several sources. More information on the NHANES DS component protocol can be found elsewhere [14,15]. For the analyses presented in this report, the specific types of products were chosen for presentation due to their high frequency of use among U.S. adults [2]. Single nutrient containing DS categories were constructed based on whether the DS contained any amount of the specific nutrient (i.e., calcium, iron, zinc, magnesium, selenium, folate, and vitamins D, C, B12, B6, K) [2,16]. DS use was also examined for three mutually exclusive product classes: multivitamin-minerals (MVM), multivitamins, and botanicals. MVM use was defined as a product containing three or more vitamins and one or more mineral counts per supplement [2]. Similarly, multivitamins were defined as vitamin combinations without minerals that were not categorized as MVM [17], and use of a botanical ingredient product was determined by the botanical count variable [2]. Further details regarding analysis methods have been described elsewhere [2,12,18].

Family income-to-poverty ratio (PIR), Supplemental Nutrition Program (SNAP) participation status, and food security were also assessed during the household interview. PIR is a measure of income that was established by the Department of Health and Human Services to represent the ratio of household income to the poverty guidelines, after adjusting for inflation and family size [19]. The poverty guidelines are updated annually and differ by geographical location (with different cutoffs for the 48 contiguous states, the District of Columbia, Puerto Rico, Alaska, and Hawaii). A PIR ≤ 130% is the cutoff to determine financial eligibility for SNAP, the largest federally funded nutrition assistance program that provides vouchers for food purchases with the objective of reducing hunger and improving the health of low-income individuals and families [20,21]. Three PIR categories were constructed for this analysis: ≤130%, 131–350%, and ≥350%. The Food Security Questionnaire was used to collect information on both SNAP participation and adult food security. SNAP participation was assessed based on information collected on whether the respondent was currently a beneficiary. Individuals classified as SNAP income-eligible nonparticipants consisted of individuals who are not currently a beneficiary of SNAP yet are financially eligible (PIR ≤ 130%) to receive SNAP benefits. Individuals classified as SNAP income-ineligible nonparticipants are individuals who are not currently a beneficiary of SNAP nor are they financially eligible to receive SNAP benefits, due to a PIR > 130%. Adult food security was assessed using 10 questions in the USDA’s Food Security Survey Module [22]. A dichotomous adult food security variable was constructed from the four options included in the module: adults who were considered to have full food security (no affirmative responses) or marginal food security (1–2 affirmative responses) were classified as food secure, while those with low food security (3–5 affirmative responses) or very low food security (6–10 affirmative responses) were classified as food insecure. 

Body mass index (BMI), obtained from height and weight measured during the health examination, was calculated as kg/m^2^. The classifications for BMI were as follows: underweight (<18.5), normal (18.5–24.9), overweight (25.0–29.9), and obese (≥30) [23]. BMI data are only available for participants who attended the mobile examination center (*n* = 10,863).

### Statistical Analysis

All statistical analyses were performed using SAS software (version 9.4; SAS Institute Inc., Cary, NC, USA) and SAS-callable SUDAAN software (version 11; Research Triangle Institute, Raleigh, NC, USA). For data obtained via the household questionnaire, all analyses were conducted using the NHANES interview weights to account for differential nonresponse and noncoverage, and to adjust for oversampling and post-stratification. In contrast, NHANES examination weights were used to account for nonresponse and oversampling in all analyses that included BMI, since that data was collected in the mobile examination center. A Taylor Series Linearization approach was used to approximate standard errors (SEs) for all estimates, and statistical comparisons of DS use were evaluated using a univariate *t* statistic. A Bonferroni-corrected *p*-value of 0.0167 was considered statistically significant.

## 3. Results

About half of all U.S. adults (52% ± 1.0 SE) took at least one DS in a 30-day period. DS use was higher among women (59%) than men (45%), and use increased linearly with age (Table 1). Specifically, older (71+ years) women had the highest prevalence of DS use (79%), while younger (19–30 years) men had the lowest (32%). Non-Hispanic whites (58%) and non-Hispanic Asians (53%) had a higher use of DS than non-Hispanic blacks (40%) or Hispanics (35%). Participants categorized as obese reported less DS use than those categorized as normal or overweight. Other differences were also evident; prevalence of use was higher among those who were former smokers (61% vs. 39%), those who had a self-reported health status of excellent or very good (58% vs. 49%), those with private health coverage (57% vs. 35%), or who typically consumed a moderate amount of alcohol (1 drink/day; 63% vs. 35%) compared to their counterparts. Patterns of MVM use generally followed these same general trends (Table 1). 

Overall DS use, type, and number of products consumed differed by income, with consistent patterns for DS use observed across all levels of income and food security and by SNAP participation. Higher income (SNAP income-ineligible and PIR ≥ 350%) and food-secure populations particularly were more likely to consume one or more DS compared to those who were less affluent (Table 2). The prevalence of DS use significantly increased in a stepwise fashion for adults across all age categories of PIR. Older adults (71+ years) remained the highest consumers of DS, specifically among the PIR ≥ 350% group (82%), while younger adults (19–30 years), predominantly those in the PIR ≤ 130% group, were the lowest DS consumers (28%). Similarly, the food insecure compared to the food secure, SNAP participants and SNAP income-eligible nonparticipants had lower DS prevalence of use than income-ineligible nonparticipants. 

The prevalence of MVM use also differed by income. SNAP income-ineligible, PIR ≥ 350%, and food-secure groups used MVMs the most. MVM use was highest among older adults (71+ years), non-Hispanic whites, and women (Table 3). Interestingly, half of older adults (71+ years) with a PIR ≥ 350% commonly took a MVM (51%), whereas patterns of MVM use were significantly lower among those with a PIR ≤ 130%. Also those with a PIR ≤ 130%, younger adults (19–30 years), and Hispanics were the least likely to take an MVM when compared to other PIR groups. Similar patterns of use were evident across food security categories; those who were food insecure (19%) had a significantly lower prevalence of MVM use when compared to their food-secure counterparts (33%). Across all poverty indicators, SNAP participants, specifically men (12%), had the lowest prevalence of MVM use, substantially lower than men who were SNAP income-ineligible nonparticipants (34%). 

MVM, multivitamin, and botanical users tended to have higher incomes than non-users. Of these three DS product categories, MVMs were the most commonly consumed DS (31%), followed by botanicals (7%) and multivitamins (6%) (data not shown). Approximately 2% of dietary supplement users take all three supplements (MVM, multivitamin, and botanical), and 8% of users commonly take both a MVM and a botanical (data not shown). About 7% of U.S. adults take a botanical supplement; botanical use is highest among older adults (71+ years; 10%), primarily older women (9%), with those over the age of 51 accounting for 20% of botanical users (data not shown). Interestingly, non-Hispanic whites were more likely to take a botanical than non-Hispanic blacks, non-Hispanic Asians, or Hispanics (data not shown). Those who were food-secure, SNAP income-eligible nonparticipants, and those who had a PIR ≥ 350% were more likely to take an MVM, multivitamin, and botanical than those who were food insecure, SNAP participants, or who had a PIR ≤ 130%, in that order (Figure S1). MVM and botanical use was significantly different between categories across all three poverty indicators. However, this was not the case with multivitamin use. Those with a PIR ≥ 350% had a significantly higher prevalence of multivitamin use than their lower income counterparts, while those with a PIR between 131–350% did not significantly differ in use of a multivitamin than those with a PIR ≤ 130%. Likewise, SNAP income-eligible nonparticipants and SNAP participants did not significantly differ in multivitamin use; however, SNAP income-ineligible nonparticipants were significantly more likely to take a multivitamin. On average, the majority of U.S. adult DS users (66%) took one or two supplements daily (Table 4). During a 30-day period, 61% of DS users took their supplements every day, 12% took them on 20–29 days, 11% took them on 10–19 days, and 15% took DS on fewer than 10 days (data not shown). Among DS users, use of products containing one or more selected vitamins ranged from 45 to 75% (Table S1). These vitamins included vitamins B-6, B-12, C, D, or K. Vitamin K use was the lowest overall (45%), while vitamin D use was the highest (75%). Likewise, between 33 and 71% of DS users took a supplement containing calcium, iron, zinc, magnesium, selenium, or folate during a 30-day period. Supplements containing iron were the least commonly consumed DS (33%), while supplements containing calcium (71%) were the most commonly consumed DS over the 30-day period. These vitamins and minerals were selected for presentation based on whether the vitamin or mineral reported was taken by at least 30% of consumers.

## 4. Discussion 

Results from this analysis indicate that over half of U.S. adults (52%) take one or more DS, particularly MVMs, and income is associated with DS use, type, and number of supplements taken. Many characteristics of DS use were also observed in other recent reports [2,12,24], such as comparable patterns of age, sex, and racial differences between U.S. population subgroups. DS use has remained relatively stable overtime. While 52% of the U.S. adult population used supplements in 2000 [25], a similar percentage of U.S. adults (52%) reported taking a supplement in the present study. Likewise, DS use among adults (≥19 years) was estimated to be 54% in 2003–2006 [2]. Similar to previous studies, use of DS in adults was also associated with characteristics associated with good health, such as lower BMIs, moderate alcohol use, abstinence from smoking, having private health insurance, and higher educational attainment [12,18,26]. This study provides additional, updated information on DS use in relationship to family income, food security and SNAP participation status. To our knowledge, this study is the first to use NHANES to provide updated information assessing the relationship between dietary supplement use and indicators of participants’ economic status in U.S. adults. 

According to the 2015 DGA Advisory Committee, food insecurity, or living without “consistent, dependable access to enough food for active healthy living” has the potential to limit an individual’s capacity to choose a healthy diet [27,28]. Although over 40 million people currently receive SNAP benefits [20], 40.6 million people live in poverty, and approximately 13% of U.S. households are food-insecure [28], suggesting that some of these persons may be at increased risk of dietary inadequacy. Adults in poor socioeconomic status have a higher prevalence of micronutrient inadequacies based on total nutrient intakes from both diet and DS [3,7]. 

Previous studies have shown that compliance with federal nutrition recommendations is especially problematic among the lower income populations [9]. In part, this may be because nutrient rich foods tend to be more expensive than lower-quality foods [29,30]. However, studies have also shown that despite having a high-income status (PIR ≥ 350%) and access to better-quality foods, some population subgroups continue to have inadequate micronutrient intakes, suggesting that the relationship between micronutrient status and income remains unclear [3]. 

The strengths and limitations of the present study should be noted. Although MVMs are the most commonly reported supplement used, no legal regulatory definition exists for MVMs [31]. Despite the self-reported nature of NHANES, DS containers and labels were seen 83% of the time by interviewers to verify accuracy. NHANES is a nationally representative survey of the U.S. noninstitutionalized population. However, the response rates for the years 2011–2012 and 2013–2014 for adults were 66% and 65%, respectively [32,33]. We cannot completely rule out the potential for self-selection bias; that is, people who are more health-conscious may have been more interested in participating in NHANES. Furthermore, given the cross-sectional nature of the data we cannot infer causality between income and DS use.

In conclusion, DS are used by over half (52%) of U.S. adults, ≥19 years; MVM supplements are the most frequently consumed supplement across all adult age groups. All of the income indicators used in this analysis were also related to the prevalence of DS use and with the type and number of products consumed. 

## Figures and Tables

**Table 1:** Estimated prevalence (%) of any dietary supplement (DS) use and multivitamin-mineral (MVM) use by demographic, anthropometric, and lifestyle characteristics among U.S. adults (≥19 years), NHANES 2011–2014 ^1,2,3^.

		Any DS			MVM		
Characteristic	*n*	Total % (SE)	Men (*n* = 5425)	Women (*n* = 5599)	Total % (SE)	Men (*n* = 5425)	Women (*n* = 5599)
Total	11,024	52.1 (1.0)	45.4 (1.1)	58.6 (1.2) *	31.2 (0.8)	28.3 (0.7) ^1^	34.0 (1.1) ^2^
Age range, years							
19–30	2284	35.5 (1.9) ^a^	31.6 (2.1)	40.0 (2.6) *	22.6 (1.5) ^a^	19.5 (1.7)	26.1(2.2) *
31–50	3686	45.2 (1.0) ^b^	38.4 (1.5)	51.7 (1.7) *	29.1 (0.9) ^b^	25.1 (1.0)	33.0 (1.4) *
51–70	3524	63.3 (1.6) ^c^	56.3 (1.8)	69.8 (1.8) *	35.4 (1.4) ^c^	34.5 (1.6)	36.2 (1.7)
≥71	1530	74.9 (1.2) ^d^	69.3 (1.7)	79.0 (1.5) *	42.7 (1.3) ^d^	40.9 (2.1)	44.0 (1.8)
Race/ethnicity	11,024						
Non-Hispanic White	4346	58.2 (1.1) ^a^	51.3 (1.3)	64.8 (1.4) *	35.7 (1.0) ^a^	32.8 (0.9)	38.5 (1.3) *
Non-Hispanic Black	2605	40.3 (1.4) ^b^	33.9 (1.8)	45.5 (1.8) *	22.6 (1.0) ^b^	20.3 (1.3)	24.6 (1.3)
Hispanic	2362	35.3 (1.1) ^c^	27.5 (1.4)	43.2 (1.5) *	19.7 (1.0) ^b^	15.3 (1.1)	24.2 (1.6) *
Non-Hispanic Asian	1388	53.5 (2.1) ^a^	47.3 (2.3)	58.9 (2.5) *	28.8 (1.6) ^c^	28.2 (1.8)	29.2 (2.0)
Educational Attainment	10,710						
Less than high school	2436	37.8 (1.1) ^a^	30.2 (1.4)	45.9 (1.5) *	20.6 (1.1) ^a^	17.7 (1.3)	23.7 (1.6) *
High school diploma/GED	2343	47.2 (1.5) ^b^	36.7 (1.8)	58.2 (2.1) *	25.2 (1.2) ^b^	19.2 (1.7)	31.6 (2.1) *
More than high school	5931	58.1 (1.1) ^c^	53.5 (1.3)	62.3 (1.4) *	36.3 (0.9) ^c^	35.0 (1.2)	37.5 (1.3)
BMI (kg/m^2^)	10,863						
<18.5	217	46.8 (5.1) ^a,b^	35.2 (7.4)	52.9 (7.2)	25.6 (4.4) ^a,b^	16.3 (6.2)	30.5 (5.3)
18.5–24.9	3220	54.2 (1.7) ^a^	44.1 (1.7)	62.5 (2.1) *	32.8 (1.6) ^a^	27.0 (1.4)	37.6 (2.1) *
25.0–29.9	3454	54.6 (1.5) ^a^	50.0 (1.8)	60.5 (1.8) *	34.0 (1.4) ^a^	31.8 (1.6)	36.8 (1.6) *
≥30	3972	48.5 (0.9) ^b^	41.5 (1.3)	54.5 (1.4) *	27.7 (0.9) ^b^	26.0 (1.3)	29.2 (1.2)
Smoking Status	10,858						
Never	6161	53.5 (1.1) ^a^	47.7 (1.6)	58.0 (1.3) *	32.5 (0.9) ^a^	30.2 (1.2)	34.3 (1.2)
Former	2484	61.0 (1.5) ^b^	53.5 (1.8)	70.8 (2.2) *	37.8 (1.6) ^b^	34.9 (1.8)	41.5 (2.3) *
Current, occasional	412	40.3 (3.0) ^c^	34.2 (3.1)	49.6 (3.6) *	23.2 (2.4) ^c^	21.7 (2.4)	25.5 (4.2)
Current, daily	1801	38.8 (1.6) ^c^	30.9 (1.6)	47.7 (2.6) *	19.9 (1.3) ^c^	15.6 (2.0)	24.7 (2.0) *
Alcohol use, drinks/day	9898						
0	3212	54.7 (1.8) ^a^	47.7 (2.0)	60.0 (2.4) *	29.5 (1.5) ^a^	26.8 (1.9)	31.6 (2.2)
1	2368	62.6 (1.3) ^b^	56.3 (2.4)	66.3 (1.6) *	39.0 (1.5) ^b^	36.9 (1.8)	40.2 (1.8)
2	2801	53.0 (1.4) ^a^	49.2 (1.7)	57.2 (2.1) *	32.0 (1.2) ^a^	30.7 (1.4)	33.5 (2.2)
≥3	1517	35.5 (2.0) ^c^	32.8 (2.1)	43.3 (3.4) *	23.4 (1.8) ^c^	22.1 (1.9)	26.9 (2.6)
Self-reported health status	9951						
Excellent or very good	3591	57.8 (1.3) ^a^	50.5 (1.8)	65.1 (1.6) *	36.7 (1.2) ^a^	32.4 (1.3)	41.0 (1.7) *
Good	4030	49.4 (1.3) ^b^	42.8 (1.5)	56.4 (1.9) *	29.3 (1.0) ^b^	27.2 (1.2)	31.5 (1.6) *
Fair or poor	2330	49.0 (1.5) ^b^	43.1 (2.3)	54.1 (1.7) *	25.0 (1.5) ^b^	24.0 (2.2)	25.9 (2.1)
Health insurance coverage	10,977						
Private	5580	57.5 (1.1) ^a^	51.1 (1.5)	63.7 (1.4) *	35.1 (1.0) ^a^	32.4 (1.1)	37.7 (1.3) *
Public	2913	53.1 (1.6) ^a^	46.8 (1.9)	58.1 (2.3) *	29.3 (1.4) ^b^	28.1 (1.8)	30.3 (2.0)
Uninsured	2484	34.7 (1.5) ^b^	28.7 (1.9)	41.8 (2.1) *	21.1 (1.1) ^c^	17.3 (1.3)	25.6 (1.9)

Abbreviations: BMI, body mass index (calculation as weight in kilograms divided by height in meters squared); SE, standard error. ^1^ Different superscript letters (a, b, c) indicate significant differences within a column at a Bonferroni corrected *p* < 0.0167, determined by using a univariate t statistic. ^2^ An asterisk “*” indicates significant differences between sex within a row at a Bonferroni corrected *p* < 0.0167, determined by using a univariate *t* statistic. ^3^ Data are presented as percentages (SE); sample size is 11,024 unless otherwise noted.

**Table 2:** Estimated prevalence (%) of dietary supplement (DS) use by selected poverty and demographic indicators, among U.S. adults, 2011–2014 ^1,2^.

	Any DS								
	Total (*n* = 11,024)	PIR			Food Security		SNAP Participation		
		PIR ≤ 130% (*n* = 3661)	131–350% (*n* = 3430)	≥350% (*n* = 3040)	Food-Insecure (*n* = 8829)	Food-Secure (*n* = 2115)	SNAP Participant (*n* = 2267)	Income-Eligible Nonparticipant (*n* = 2030)	Income-Ineligible Nonparticipant (*n* = 5963)
**All**	52.1 (1.0)	38.6 (1.5) ^a^	50.3 (1.0) ^b^	63.5 (1.3) ^c^	36.4 (1.7) ^a^	55.1 (1.0) ^b^	32.1 (1.3) ^a^	44.4 (1.9) ^b^	59.0 (0.9) ^c^
**Sex**									
Men	45.4 (1.1)	30.2 (1.4) ^a^	41.9 (1.4) ^b^	58.3 (1.6) ^c^	29.1 (2.2) ^a^	48.3 (1.3) ^b^	23.7 (1.5) ^a^	34.1 (2.0) ^b^	52.8 (1.2) ^c^
Women	58.6 (1.2)	45.7 (2.0) ^a^	58.3 (1.6) ^b^	69.1 (1.7) ^c^	43.2 (1.9) ^a^	61.6 (1.2) ^b^	38.9 (1.8) ^a^	54.1 (2.5) ^b^	65.2 (1.2) ^c^
**Age**									
19–30 years	35.8 (1.9)	27.6 (2.5) ^a^	36.3 (2.5) ^b^	46.3 (3.7) ^b^	30.7 (3.1)	36.9 (2.1)	22.0 (2.2) ^a^	32.0 (2.5) ^b^	42.2 (2.6) ^b^
31–50 years	45.2 (1.0)	34.7 (2.0) ^a^	43.4 (1.8) ^b^	55.2 (1.5) ^c^	34.8 (2.3) ^a^	47.6 (1.1) ^b^	26.3 (1.8) ^a^	41.7 (3.1) ^b^	51.7 (1.2) ^c^
51–70 years	63.3 (1.6)	47.7 (1.8) ^a^	57.0 (2.3) ^b^	73.9 (1.9) ^c^	41.6 (3.1) ^a^	66.2 (1.6) ^b^	44.1 (2.4) ^a^	52.7 (2.5) ^b^	68.5 (1.6) ^c^
71+ years	74.9 (1.2)	66.3 (3.1) ^a^	75.0 (1.7) ^a^	82.1 (2.1) ^c^	59.8 (4.6) ^a^	75.7 (1.2) ^b^	59.5 (4.2) ^a^	69.3 (4.5) ^a,b^	78.7 (1.3) ^b^
**Race**									
Non-Hispanic White	58.2 (1.1)	44.9 (1.7) ^a^	55.3 (1.7) ^b^	66.2 (1.3) ^c^	41.5 (2.8) ^a^	60.4 (1.0) ^b^	35.9 (2.4) ^a^	51.6 (2.2) ^b^	62.9 (1.1) ^c^
Non-Hispanic Black	40.3 (1.4)	33.0 (2.2) ^a^	42.0 (1.9) ^b^	49.2 (3.0) ^b^	30.7 (2.0) ^a^	43.0 (1.5) ^b^	30.9 (2.1) ^a^	37.4 (3.2) ^a,b^	46.5 (2.1) ^b^
Hispanic	35.3 (1.1)	29.6 (2.1) ^a^	36.4 (1.9) ^a^	49.4 (2.9) ^b^	31.7 (2.2)	37.0 (1.2)	26.1 (2.0) ^a^	32.6 (2.8) ^a^	42.3 (1.7) ^b^
Non-Hispanic Asian	53.5 (2.1)	43.3 (4.3) ^a^	48.7 (3.5) ^a^	62.6 (2.7) ^b^	38.7 (6.0) ^a^	54.6 (2.2) ^b^	41.3 (6.5) ^a,b^	45.6 (4.1) ^a^	57.1 (2.3) ^b^

Abbreviations: PIR, poverty–income ratio; SNAP, Supplemental Nutrition Assistance Program. ^1^ Different superscript letters (a, b, c) indicate significant differences within a row at a Bonferroni corrected *p* < 0.0167, determined by using a univariate *t* statistic. Missing superscripts indicate that the difference between groups within a category was not statistically significant. ^2^ Data are presented as percentages (SE); sample size is 11,024 unless otherwise noted.

**Table 3:** Estimated prevalence (%) of multivitamin-mineral (MVM) use by selected poverty and demographic indicators, among U.S. adults, 2011–2014 ^1,2^.

	MVM								
	Total (*n* = 11,024)	PIR			Food Security		SNAP Participation		
		PIR ≤ 130% (*n* = 3661)	131–350% (*n* = 3430)	≥350% (*n* = 3040)	Food-Insecure (*n* = 8829)	Food-Secure (*n* = 2115)	SNAP Participant (*n* = 2267)	Income-Eligible Nonparticipant (*n* = 2030)	Income-Ineligible Nonparticipant (*n* = 5963)
**All**	31.2 (0.8)	20.5 (1.2) ^a^	29.1 (1.0) ^b^	40.7 (1.2) ^c^	18.9 (1.6) ^a^	33.5 (0.8) ^b^	16.4 (1.0) ^a^	24.6 (1.5) ^b^	36.6 (0.9) ^c^
**Sex**									
Men	28.3 (0.7)	15.5 (1.2) ^a^	25.4 (1.1) ^b^	38.8 (1.3) ^c^	15.5 (1.8) ^a^	30.6 (0.8) ^b^	12.4 (1.3) ^a^	18.1 (1.5) ^b^	34.1 (0.9) ^c^
Women	34.0 (1.1)	24.6 (1.7) ^a^	32.6 (1.4) ^b^	42.9 (1.8) ^c^	22.1 (1.8) ^a^	36.3 (1.1) ^b^	19.5 (1.3) ^a^	30.6 (2.2) ^b^	39.1 (1.2) ^c^
**Age**									
19–30 years	22.6 (1.5)	15.4 (1.8) ^a^	22.4 (1.5) ^a,b^	32.4 (3.5) ^b^	17.7 (2.6)	23.9 (1.8)	12.8 (1.6) ^a^	18.5 (2.0) ^b^	27.8 (2.3) ^c^
31–50 years	29.1 (0.9)	19.5 (1.4) ^a^	28.0 (1.6) ^b^	37.9 (1.9) ^c^	18.6 (2.4) ^a^	31.5 (1.1) ^b^	14.1 (1.3) ^a^	23.8 (1.9) ^b^	35.0 (1.3) ^c^
51–70 years	35.4 (1.4)	23.0 (2.1) ^a^	29.1 (2.1) ^a^	44.5 (1.9) ^b^	19.5 (2.6) ^a^	37.6 (1.5) ^b^	21.7 (2.1) ^a^	26.2 (2.6) ^a^	39.3 (1.7) ^b^
71+ years	42.7 (1.3)	34.4 (2.6) ^a^	42.3 (2.6) ^a,b^	50.9 (3.1) ^b^	27.3 (6.0) ^a^	43.7 (1.4) ^b^	21.7 (3.9) ^a^	40.1 (3.7) ^b^	46.7 (1.8) ^b^
**Race**									
Non-Hispanic White	35.7 (1.0)	24.3 (2.0) ^a^	32.4 (1.6) ^b^	42.9 (1.4) ^c^	22.1 (2.9) ^a^	37.5 (1.0) ^b^	18.5 (2.0) ^a^	28.9 (2.3) ^b^	39.6 (1.1) ^c^
Non-Hispanic Black	22.6 (1.0)	17.0 (2.1) ^a^	24.0 (1.4) ^b^	30.9 (2.0) ^c^	15.5 (2.0) ^a^	24.7 (1.1) ^b^	15.3 (1.8) ^a^	21.7 (3.3) ^a,b^	27.5 (1.1) ^b^
Hispanic	19.7 (1.0)	15.5 (1.8) ^a^	20.7 (1.3) ^a^	31.1 (2.7) ^b^	16.0 (2.0) ^a^	21.2 (1.0) ^b^	13.1 (1.5) ^a^	17.7 (2.4) ^a^	25.1 (1.3) ^b^
Non-Hispanic Asian	28.8 (1.6)	19.8 (3.1) ^a^	25.4 (2.7) ^a^	36.5 (2.1) ^b^	16.0 (4.3) ^a^	29.7 (1.6) ^b^	15.9 (4.0) ^a^	21.8 (3.6) ^a^	32.4 (1.9) ^b^

Abbreviations: PIR, poverty–income ratio; SNAP, Supplemental Nutrition Assistance Program. ^1^ Different superscript letters (a, b, c) indicate significant differences within a row at a Bonferroni corrected *p* < 0.0167, determined by using a univariate *t* statistic. Missing superscripts indicate that the difference between groups within a category was not statistically significant. ^2^ Data are presented as percentages (SE); sample size is 11,024 unless otherwise noted.

**Table 4:** Estimated prevalence (%) of dietary supplement use by number of dietary supplements taken and selected poverty indicators among U.S. adult supplement users, 2011–2014 ^1,2^.

	Total (*n* = 5375)	PIR			Food Security		SNAP Participation		
		PIR ≤ 130% (*n* = 1438)	131–350% (*n* = 1678)	≥350% (*n* = 1867)	Food-Insecure (*n* = 769)	Food-Secure (*n* = 4573)	SNAP Participant (*n* = 755)	Income-Eligible Nonparticipant (*n* = 913)	Income-Ineligible Nonparticipant (*n* = 3358)
**Number of supplements**									
1	42.7 (1.1)	53.9 (2.3) ^a^	43.1 (1.5) ^b^	37.2 (1.5)^c^	58.7 (2.7) ^a^	40.8 (1.2) ^b^	57.8 (2.1) ^a^	50.8 (2.8) ^a^	39.1 (1.1) ^b^
2	22.9 (0.8)	19.8 (1.1) ^a^	22.3 (1.3) ^a,b^	25.3 (1.8) ^b^	19.5 (1.8)	23.3 (0.9)	19.7 (1.7) ^a^	20.2 (1.3) ^a,b^	24.0 (1.0) ^b^
3	14.6 (0.5)	11.8 (1.0) ^a^	12.8 (1.2) ^a,b^	16.3 (1.0) ^b^	10.0 (1.5) ^a^	15.1 (0.5) ^b^	9.9 (1.6) ^a^	12.9 (1.5) ^a,b^	15.1 (0.6) ^b^
4	7.6 (0.5)	5.4 (0.1) ^a^	7.6 (0.7) ^a,b^	8.9 (0.8) ^b^	4.3 (0.8) ^a^	8.0 (0.6) ^b^	4.3 (0.8) ^a^	6.5 (1.4) ^a,b^	8.5 (0.6) ^b^
5 or more	12.2 (0.7)	9.1 (1.3) ^a^	14.1 (1.1) ^b^	12.6 (1.1) ^a,b^	7.3 (1.3) ^a^	12.8 (0.7) ^b^	8.4 (1.6) ^a^	9.5 (1.4) ^a^	13.1 (0.7) ^b^

Abbreviations: MVM, multivitamin-mineral; PIR, poverty–income ratio; SNAP, Supplemental Nutrition Assistance Program. ^1^ Different superscript letters (a, b, c) indicate significant differences within a row at a Bonferroni corrected *p* < 0.0167, determined by using a univariate *t* statistic. Missing superscripts indicate that the difference between groups within a category was not statistically significant. ^2^ Data are presented as percentages (SE); sample size is 5375 unless otherwise noted.

## Supplementary Materials

The following are available online at http://www.mdpi.com/2072-6643/10/8/1114/s1, Table S1: Estimated prevalence (%) of dietary supplement use by of type of dietary supplement and selected poverty indicators among U.S. adult supplement users, 2011–2014, Figure S1: Estimated prevalence (%) of multivitamin-mineral (MVM), multivitamin, and botanical use by selected poverty indicators among U.S. adults, 2011–2014.

## References

[1] U.S. Department of Health and Human Services 2015–2020 Dietary Guidelines for Americans. https://health.gov/dietaryguidelines/2015/guidelines/.

[2] Bailey R.L., Gahche J.J., Lentino C.V., Dwyer J.T., Engel J.S., Thomas P.R., Betz J.M., Sempos C.T., Picciano M.F. (2011). Dietary supplement use in the United States, 2003–2006. J. Nutr..

[3] Bailey R.L., Akabas S.R., Paxson E.E., Thuppal S.V., Saklani S., Tucker K.L. (2017). Total usual intake of shortfall nutrients varies with poverty among U.S. adults. J. Nutr. Educ. Behav..

[4] Trends in prevalence rates of food insecurity and very low food security in U.S. Households, 1995–2016. https://www.ers.usda.gov/topics/food-nutrition-assistance/food-security-in-the-us/key-statistics-graphics.aspx#trends.

[5] Bishaw A. (2013). Poverty: 2000 to 2012.

[6] Semega J.L., Fontenot K.R., Kollar M.A. (2017). Income and Poverty in the United States: 2016.

[7] Blumberg J.B., Frei B., Fulgoni V.L., Weaver C.M., Zeisel S.H. (2017). Contribution of dietary supplements to nutritional adequacy by socioeconomic subgroups in adults of the United States. Nutrients.

[8] Kant A.K., Graubard B.I. (2007). Secular trends in the association of socio-economic position with self-reported dietary attributes and biomarkers in the US population: National Health and Nutrition Examination Survey (NHANES) 1971–1975 to NHANES 1999–2002. Public Health Nutr..

[9] Monsivais P., Aggarwal A., Drewnowski A. (2011). Following federal guidelines to increase nutrient consumption may lead to higher food costs for consumers. Health Aff..

[10] Zipf G., Chiappa M., Porter K.S. (2013). National Health and Nutrition Examination Survey: Plan and Operations, 1999–2010.

[11] Otten J.J., Hellwig J.P., Meyers L.D. (2006). Dietary Reference Intakes: The Essential Guide to Nutrient Requirements.

[12] Gahche J.J., Bailey R.L., Potischman N., Dwyer J.T. (2017). Dietary supplement use was very high among older adults in the United States in 2011–2014. J. Nutr..

[13] MacLean R.R., Cowan A., Vernarelli J.A. (2018). More to gain: Dietary energy density is related to smoking status in U.S. adults. BMC Public Health.

[14] National Center for Health Statistics National Health and Nutrition Examination Survey 2011–2012 Data Documentation, Codebook, and Frequencies: Dietary Supplement Use 30-day-Total Dietary Supplements.

[15] National Center for Health Statistics National Health and Nutrition Examination Survey 2013–2014 Data Documentation, Codebook, and Frequencies: Dietary Supplement Use 30-day-Total Dietary Supplements.

[16] Ervin R.B., Wright J.D., Kennedy-Stephenson J. (1999). Use of Dietary Supplements in the United States, 1988-1994.

[17] Bailey R.L., Fakhouri T.H., Park Y., Dwyer J.T., Thomas P.R., Gahche J.J., Miller P.E., Dodd K.W., Sempos C.T., Murray D.M. (2015). Multivitamin-mineral use is associated with reduced risk of cardiovascular disease mortality among women in the United States. J. Nutr..

[18] Bailey R.L., Gahche J.J., Miller P.E., Thomas P.R., Dwyer J.T. (2013). Why us adults use dietary supplements. JAMA Internal Med..

[19] U.S. Department of Health and Human Services The Poverty Guidelines Updated Periodically in the Federal Register by the U.S. Department of Health and Human Services under the Authority of 42 U.S.C. 9902(2).

[20] Gundersen C. (2013). Food insecurity is an ongoing national concern. Adv. Nutr..

[21] U.S. Department of Agriculture (2012). Building a Healthy America: A Profile of the Supplemental Nutrition Assistance Program.

[22] U.S. Adult Food Security Survey Module: Three-stage Design, with Screeners. https://www.ers.usda.gov/media/8279/ad2012.pdf.

[23] Goodwin S. (2000). Practical Guide: Identification, Evaluation, and Treatment of Overweight and Obesity in Adults.

[24] Gahche J., Bailey R., Burt V., Hughes J., Yetley E., Dwyer J., Picciano M.F., McDowell M., Sempos C. (2011). Dietary supplement use among U.S. adults has increased since NHANES III (1988–1994). NCHS Data Brief.

[25] Kantor E.D., Rehm C.D., Du M., White E., Giovannucci E.L. (2016). Trends in dietary supplement use among U.S. adults from 1999–2012. JAMA.

[26] Rock C.L. (2007). Multivitamin-multimineral supplements: Who uses them?. Am. J. Clini. Nutr..

[27] Dietary Guidelines Advisory Committee (2015). Scientific Report of the 2015 Dietary Guidelines Advisory Committee: Advisory Report to the Secretary of Health and Human Services and Secretary of Agriculture.

[28] Coleman-Jensen A., Rabbitt M.P., Gregory C.A., Singh A. (2017). Household Food Security in the United States in 2016.

[29] Drewnowski A., Rehm C.D. (2015). Socioeconomic gradient in consumption of whole fruit and 100% fruit juice among us children and adults. Nutr. J..

[30] Darmon N., Drewnowski A. (2015). Contribution of food prices and diet cost to socioeconomic disparities in diet quality and health: A systematic review and analysis. Nutr. Rev..

[31] Blumberg J.B., Bailey R.L., Sesso H.D., Ulrich C.M. (2018). The evolving role of multivitamin/multimineral supplement use among adults in the age of personalized nutrition. Nutrients.

[32] Unweighted Response Rates for NHANES 2011–2012 by Age and Gender. https://wwwn.cdc.gov/nchs/data/nhanes3/ResponseRates/rrt1112.pdf.

[33] Unweighted Response Rates for NHANES 2013–2014 by Age and Gender. https://wwwn.cdc.gov/nchs/data/nhanes3/ResponseRates/2013_2014_response_rates.pdf.

