# Supplementary material for: Dietary Supplement Use Differs by Socioeconomic and Health-Related Characteristics among U.S. Adults, NHANES 2011–2014
Source: Nutrients. 2018 Aug 17;10(8):1114. doi: 10.3390/nu10081114 (PMC6116059; doi:10.3390/nu10081114)
Supplement: Supplementary file 1 [file nutrients-10-01114-s001.pdf]

nutrients

MDPI

Table S1. Estimated prevalence (%) of dietary supplement use by of type of dietary supplement and selected poverty indicators among U.S. Adult supplement users, 2011-2014[1,2]

	Total (n=5,375)	PIR			Food security		SNAP		
		**PIR≤130% (n=1,438)**	**131-350% (n=1,678)**	**≥350% (n=1,867)**	**Food-insecure (n=769)**	**Food-secure (n=4,573)**	**SNAP Participant (n=755)**	**Income-eligible nonparticipant (n=913)**	**Income-ineligible nonparticipant (n=3,358)**
**Type**									
**Calcium**	70.7 (1.0)	63.2 (2.5)[a]	70.6 (1.7)[a,b]	73.8 (1.1)[b]	62.8 (2.9)[a]	71.7 (1.0)[b]	61.4 (2.9)[a]	64.3 (2.3)[a]	73.0 (1.0)[b]
**Iron**	32.6 (1.1)	34.9 (1.8)	32.4 (1.5)	32.0 (1.6)	34.8 (2.4)	32.3 (1.1)	30.9 (2.9)	35.5 (1.8)	32.4 (1.3)
**Zinc**	57.8 (1.0)	51.1 (2.1)[a]	57.1 (1.8)[b]	61.1 (1.5)[b]	49.2 (3.3)[a]	58.9 (1.0)[b]	48.0 (2.9)[a]	54.0 (2.3)[a,b]	59.9 (1.1)[b]
**Magnesium**	56.0 (1.0)	47.4 (2.0)[a]	54.7 (1.7)[b]	60.4 (1.5)[b]	48.3 (2.6)[a]	57.0 (0.9)[b]	44.8 (2.7)[a]	49.9 (2.5)[a]	58.6 (1.0)[b]
**Selenium**	50.1 (0.9)	41.6 (2.0)[a]	49.4 (1.6)[b]	54.1 (1.3)[b]	40.7 (3.1)[a]	51.4 (0.9)[b]	38.5 (2.5)[a]	44.8 (2.3)[a]	52.6 (1.0)[b]
**Folate**	61.3 (0.9)	53.5 (2.1)[a]	58.5 (1.6)[a]	66.6 (1.3)[b]	52.6 (2.9)[a]	62.5 (0.8)[b]	50.7 (2.7)[a]	56.0 (2.0)[a]	63.8 (0.9)[b]
**Vitamin D**	75.5 (0.8)	65.7 (2.0)[a]	72.8 (1.3)[b]	81.2 (1.4)[c]	64.2 (1.9)[a]	76.8 (0.8)[b]	66.0 (2.5)[a]	67.0 (2.2)[a]	78.1 (0.9)[b]
**Vitamin C**	69.4 (0.9)	61.9 (2.1)[a]	69.8 (1.5)[b]	72.7 (1.2)[b]	61.2 (3.4)[a]	70.4 (0.8)[b]	57.0 (2.8)[a]	65.9 (2.1)[b]	71.9 (1.0)[c]
**Vitamin B12**	66.1 (0.8)	60.1 (1.9)[a]	64.4 (1.7)[a]	70.1 (1.2)[b]	58.1 (2.7)[a]	67.1 (0.9)[b]	56.9 (2.3)[a]	62.9 (2.1)[a,b]	68.2 (1.0)[b]
**Vitamin B6**	62.0 (0.9)	55.2 (2.2)[a]	60.2 (1.6)[a]	66.2 (1.3)[b]	53.5 (2.9)[a]	63.2 (0.9)[b]	54.2 (2.8)[a]	57.3 (2.1)[a]	64.1 (1.0)[b]
**Vitamin K**	45.4 (1.0)	38.5 (1.9)[a]	44.6 (1.5)[b]	48.7 (1.5)[b]	37.8 (3.1)[a]	46.4 (1.1)[b]	35.1 (2.3)[a]	41.4 (2.3)[a]	47.5 (1.2)[b]

Abbreviations: PIR, poverty-income ratio; SNAP, Supplemental Nutrition Assistance Program.

[1] Different superscript letters (a,b,c) indicate significant differences within a row at a Bonferroni corrected p<0.0167, determined by using a univariate t statistic. Missing superscripts indicate that the difference between groups within a category was not statistically significant.

[2] Data are presented as percentages (SE); sample size is 5375 unless otherwise noted.

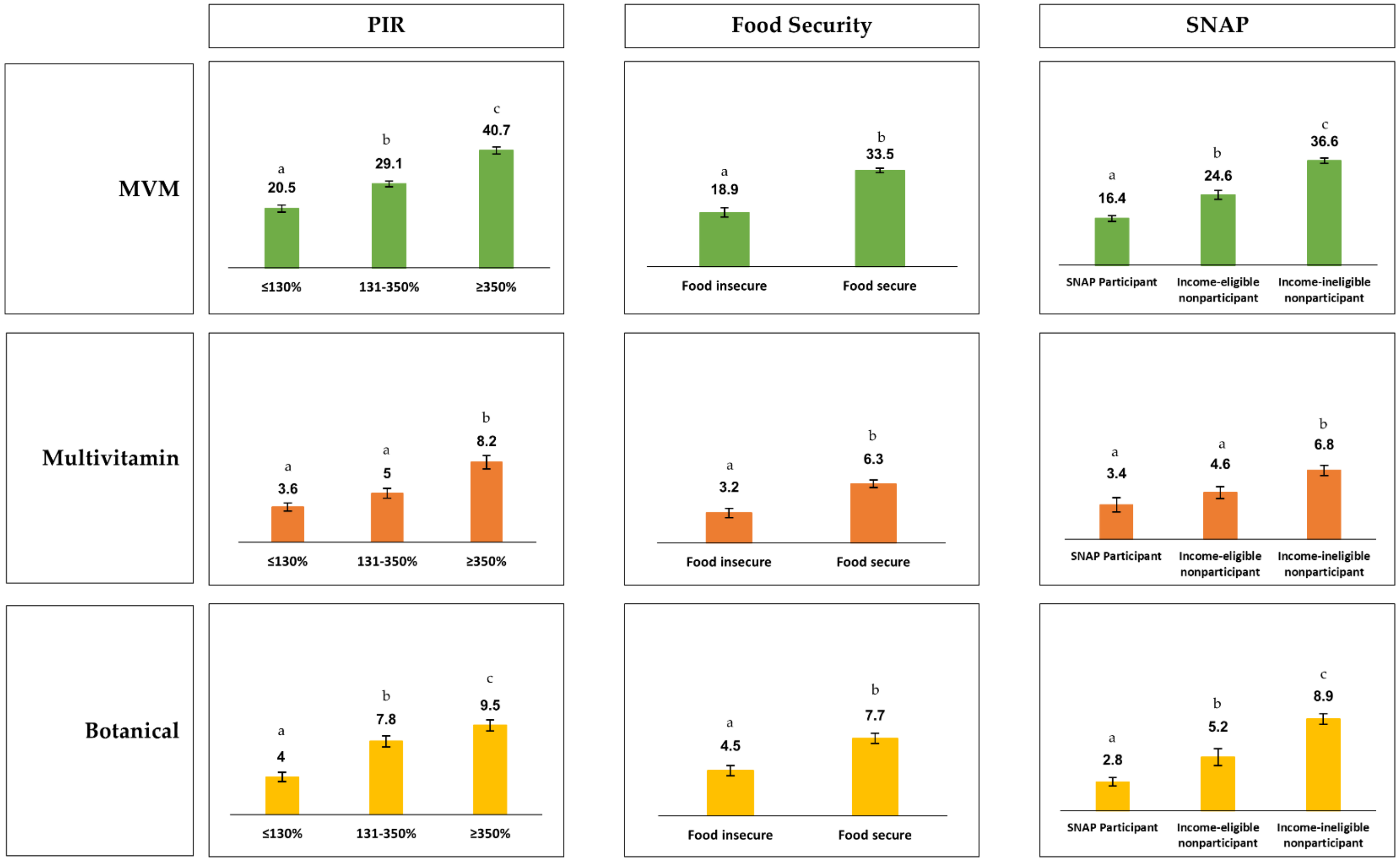

Figure S1. Estimated prevalence (%) of MVM, multivitamin, and botanical use by selected poverty indicators among U.S. Adults, 2011-2014[1,2]

Abbreviations: MVM, multivitamin-minerals; PIR, poverty-income ratio; SNAP, Supplemental Nutrition Assistance Program.

[1] Different superscript letters (a,b,c) indicate significant differences within a row at a Bonferroni corrected $p<0.0167$, determined by using a univariate t statistic.

[2] Data are presented as percentages (SE); sample size is 11 024 unless otherwise noted.
